# PAL: A Framework for Physically Assisted Learning Through Design and Exploration With a Haptic Robot Buddy

**DOI:** 10.3389/frobt.2021.700465

**Published:** 2021-09-24

**Authors:** Soheil Kianzad, Guanxiong Chen, Karon E. MacLean

**Affiliations:** ^1^ SPIN Lab, Department of Computer Science, University of British Columbia, Vancouver, BC, Canada; ^2^ Department of Electrical and Computer Engineering, University of British Columbia, Vancouver, BC, Canada

**Keywords:** educational robotics, experiential learning, haptic force feedback, interactive drawing, physically assisted learning

## Abstract

Robots are an opportunity for interactive and engaging learning activities. In this paper we consider the premise that haptic force feedback delivered through a held robot can enrich learning of science-related concepts by building physical intuition as learners design experiments and physically explore them to solve problems they have posed. Further, we conjecture that combining this rich feedback with pen-and-paper interactions, *e.g.*, to sketch experiments they want to try, could lead to fluid interactions and benefit focus. However, a number of technical barriers interfere with testing this approach, and making it accessible to learners and their teachers. In this paper, we propose a framework for Physically Assisted Learning based on stages of experiential learning which can guide designers in developing and evaluating effective technology, and which directs focus on how haptic feedback could assist with *design* and *explore* learning stages. To this end, we demonstrated a possible technical pathway to support the full experience of designing an experiment by drawing a physical system on paper, then interacting with it physically after the system recognizes the sketch, interprets as a model and renders it haptically. Our proposed framework is rooted in theoretical needs and current advances for experiential learning, pen-paper interaction and haptic technology. We further explain how to instantiate the PAL framework using available technologies and discuss a path forward to a larger vision of physically assisted learning.

## 1 Introduction

The learning of topics once delivered in physical formats, like physics and chemistry labs, has moved into digital modalities for reasons from pragmatics (cost, maintenance of setups, accessibility, remote delivery) to pedagogy (topic versatility, personalized learning, expanded parameter space including the physically impossible). Much is thereby gained. However, typically accessed as graphical user interfaces with mouse/keyboard input, these environments have lost physical interactivity: learners must grasp physical concepts in science and math through disembodied abstractions which do little to help develop physical intuition.

Physically interactive robots coupled with an interactive virtual environment (VE) offer an alternative way for students to encounter, explore and collaboratively share and build on knowledge. While contemporary technology and learning theories have not yet delivered a robot system sufficiently versatile to support a wide range of learning needs and environments, we can nevertheless propose and separately evaluate design dimensions that a haptic robot and accompanying interactive VE enables. The objective of this paper is to facilitate the design and assessment of this new class of learning technology by articulating its requirements *via* a framework.

Experiential learning theorist Kolb (1984) posits a four-phase cycle that learners ideally repeat iteratively: concrete experience (CE), reflective observation (RO), abstract conceptualization (AC), and active experimentation (AE).

In this paper we focus on how a haptic robot might be engaged in the stages of this cycle which naturally lend themselves to physical manipulation: **active experimentation**, through *designing* a virtual experimentation environment suitable for a question they have, and **concrete experience**, through *exploring* the environment they configured.

### 1.1 A Vision for Physically Assisted Learning: A Sketch-Based Design-Explore Cycle

The ability to draw a model, then feel it (active experimentation around an idea, then associated concrete experience of it—forming and testing a hypothesis) may be key to elevating interactive sketching to experiential learning. When exploring, learners can extend their understanding of a domain of knowledge by physically interacting with a virtual model—making abstract concepts more accessible, and approachable in new ways. When they are designing, physicalized digital constraints combined with sketch-recognition intelligence can help them to expeditiously express their thoughts by sketching to the system, with the added benefit of representing the resulting model to a co-learner. Finally, exploring one’s own designs now becomes a holistic cycle: the learner challenges their knowledge by dynamically posing their own questions and mini-experiments as well as others’ by designing models, then reflecting on the outcome of interacting with it.

As a concrete example: to “play with” the dynamics of a physical system (*e.g.*, a mass-spring oscillation), a learner is assisted by a force-feedback-enabled drawing stylus to sketch the system on an arbitrary surface. The system recognizes the drawn ink as, say, a mass connected to a ground through a spring. Using the same stylus, the learner can then “grab” the drawn mass and pull on it. To test a question about parallel versus series spring networks, they can mentally predict then quickly draw the two cases and physically compare the result. Similarly, they could test relative oscillatory frequencies by extending the spring then “releasing” it. By writing in a new spring constant (“K = 2”) they can modify the spring constant. The same process can be applied in other domains, such as in designing-to-explore an electronic or fluid circuit, and to improvisationally testing equations defining system properties. This use case (Figure 6) and others are implemented and elaborated later in this paper.

### 1.2 Technical Challenges and Ways Around Them

Aspects of the AE and CE experiential learning stages have been studied and validated in isolation using tangible user interfaces, robots and haptic devices, and the results underscore the general promise of this approach (Zacharia et al., 2012; Magana and Balachandran, 2017; Radu et al., 2021). However, few systems support physicalized interaction in both stages, far less fluid transition between them.

This is at least partially due to the technical difficulties of working with present-day versions of these technologies. For example, conventional grounded force-feedback haptic systems can theoretically support VE creation and interaction, but in practice, they require extensive time and expertise not just to create but even to apply small variants in learning purpose, which often is unavailable in a school setting. Their expense, limited-size and desk-tethered workspaces and single-user nature preclude mobility and collaboration and tend to be too high-cost and require significant technical support. Other robot technologies are mobile and collaboration-friendly, but do not convey physical forces—e.g. a robot puck with which a user can control tokens on a graphical screen.

However, a handheld force-feedback tool that combines a spectrum of autonomy with physical interaction can potentially overcome these technical limitations: *e.g.*, a robotic pen which can assist a learner in navigating concepts of physics and math by conveying physical forces modeled by an environment drawn by its holder. Technically, this system must read and understand the user’s sketches and notations, translate them into a VE and associated parameterized physical models, then animate this environment mechanically with a physics engine rendered through a suitable force-feedback display—ideally with the same handheld tool with which they drew the environment. A haptic device in the general form of a handheld, self-propelled and high-bandwidth robot can generate untethered, screen-free experiences that encourage collaboration.

This concept is technically feasible today without any intrinsically high-cost elements, with the haptic pen itself fully demonstrated (Kianzad and MacLean, 2018; Kianzad et al., 2020), but significant engineering remains to translate innovations in sketch recognition from other technical domains and integrate them into a full-functioned, low-latency robotic system. Our purpose in this paper is to consider the potential of this approach based on related technology elements as a proxy for a future integrated system which we know is possible to build if proven worthwhile.

### 1.3 Approach and Contributions

We have designed support based on a theory of activities that has been shown to lead to effective learning, and require this support to meet usability principles suggested by the theory. For example, the cyclical nature of Kolb (1984) et al’s learning cycle directs us to minimize cognitive and procedural friction in performing and moving between important cycle activities. Unfettered designing and exploring implies comfortable workspace size and natural command-and-control functions that transfer easily from a student’s existing experience—*e.g.*, pen-and-paper diagramming, nomenclature consistent with how they are taught, direct application of parameters, etc. They should not have to switch tools when they switch stages. Meanwhile, their work should be easily visible in a way that teachers and co-learners can see what they are doing and effectively collaborate in their experience (Asselborn et al., 2018; Khodr et al., 2020; Radu et al., 2021).

#### 1.3.1 Getting to Confidence that it Could Work

The scope of this paper is to identify and solve technical obstacles to the instantiation of the theoretically based PAL framework, focusing on the gap in previous work: the connection between physically supported design and explore learning activity, in the form of theoretical rationale and technical proof-of-concept. We need to ensure that the concept’s non-trivial realization is feasible, given obstacles ranging from stroke recognition to haptic rendering algorithm and availability of a haptic display with suitable capability and performance.

Only with this evidence will it will be ready to (beyond our present scope) optimize for usability; and thence to evaluate for the pedagogical value of adding physical expression and fluidity to the explore-design-explore cycle. Given the complex and individual process of learning, this will require a sequence of user studies to convincingly validate the framework and its impact on learning gain, as well as generalizablity across multiple platforms.

#### 1.3.2 Guiding Support and Assessing Potentials With an Experiential Learning Framework

**FIGURE 1:**
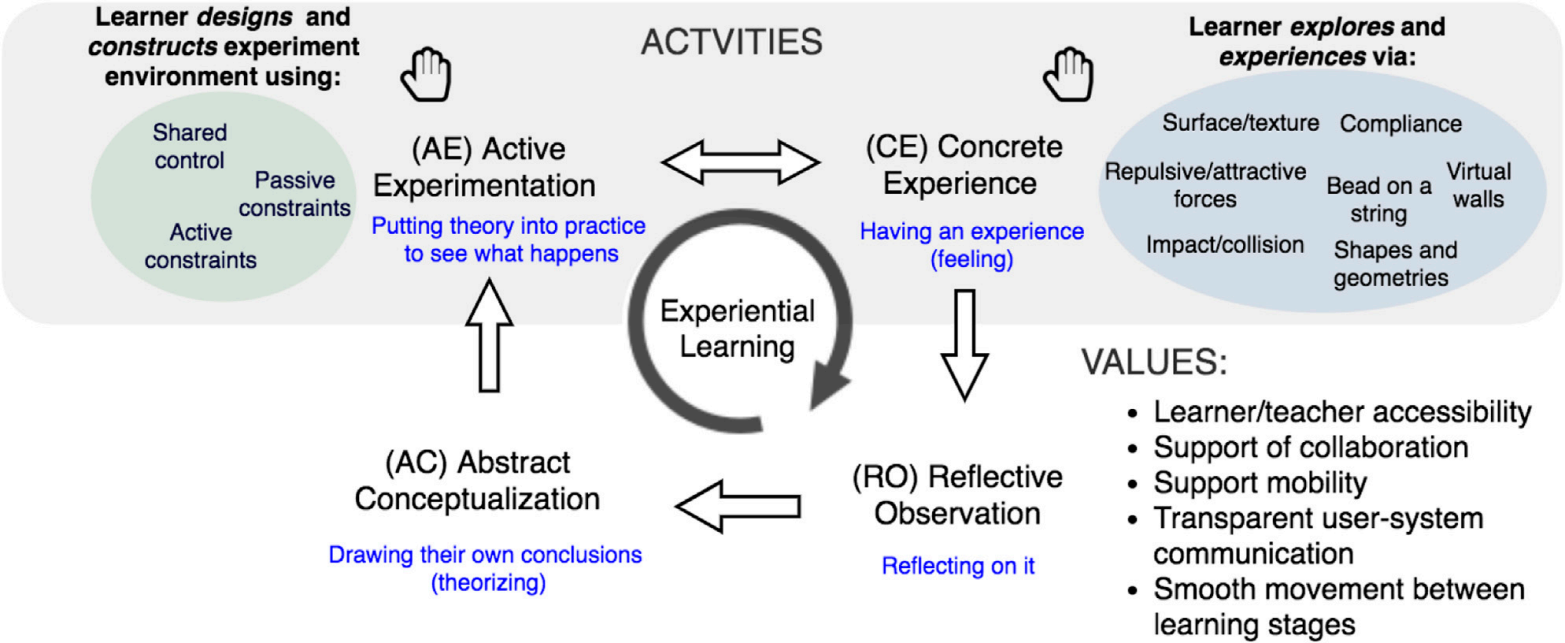
The PAL Framework. Physically Assisted Learning interactions haptically adapt stages of experiential learning from Kolb (1984)’s general framework, with some added features from Honey and Mumford (2000). Hands-on Active Experimentation and Concrete Experience are most amenable to haptic augmentation, enriching the more purely cognitive Reflective Observation and Abstract Conceptualization.

We propose a **Physically-Assisted Learning (PAL)** framework through which we can systematically compare different candidate technologies’ potentials in *unlocking key activities and values* (Figure 1). Through the PAL lens, we view learning *via* the physically supported **activities** of *designing (AE)* and *exploring (CE)*; and assess platforms against key cross-cutting **values** of *learner/teacher accessibility* (Özgür et al., 2017a), support of *collaboration*, untethered (Kianzad and MacLean, 2019), screen-free *mobility*, *transparent* user-system communication (Romat et al., 2019), and *seamless transitioning* between learning stages.

We are using PAL as a tool to understand the impact of device attributes on learning strategies and outcomes, as well as collaborative effectiveness, self-efficacy, creativity, and performance in drawing and design.

Throughout the paper, we will relate needs, technical challenges and approaches to this framework, and consider how the candidate technologies stack up on its values under the two activities of focus.

#### 1.3.3 We Contribute

1) **The Physically Assisted Learning (PAL) framework** which can 1) conceptually and constructively guide the design of haptic science-learning support; and 2) lead directly to articulation of special requirements for *explore*-type contexts like learning, including fluid access to large ranges of model structure and parameterization.
2) **Demonstrations** of 1) means of addressing these needs, for *designing* with innovative application of hand-stroke recognition, and for *exploring* through haptic rendering with a control approach not available in open libraries (namely passivity control); and 2) a technical proof-of-concept system in which *designing* and *exploring* are haptically linked: a user can draw and then feel a virtual environment.
3) **A path forward:** An account of technical considerations, challenges and possible approaches to fully realize this paradigm.

## 2 Background

We introduce past work related to the idea of physicalizing digital manipulatives, relevant classes of haptic force feedback technology, challenges in bringing this kind of technology into education environments, and ways in which haptics have been used for related activities of designing and exploring.

### 2.1 Adding Physicality to Digital Manipulatives *via* Robots

***Physical manipulatives*** are objects that aid learners in perceiving physics and math concepts by manipulating them, such as the pattern blocks, coloured chips, and coins used in early childhood education to engage learners in hands-on activities. ***Digital manipulatives*** (***DMs***) are physical objects with computational and communication capabilities that can promote different types of thinking in children by engaging them in playing and building. The history of using DMs for education dates to the early 70–80 s in several works from MIT Media Lab’s Tangible Media and Epistemology and Learning groups and the Artificial Intelligence Lab. Among them, projects such as Floor Turtle, Graphical Logo, LEGO Mindstorms, Crickets, and Curlybot introduced engaging environments to develop new approaches to thinking about mathematical concepts with encouraging results (Papert, 1980; Resnick et al., 1998; Frei et al., 2000).

***Robots*** are a class of DMs that use motion along with other visual or audio cues to express information. Children can program robots and therefore observe and experience how defining a set of rules results in intentional behaviours in them. This also gives them the freedom to decide what the robot is, based on how the robot behaves. This flexibility potentially helps learners to use the robot as a probe to explore many learning concepts in different contexts (Resnick, 1998).

Haptics can empower digital manipulatives by expanding the imagination beyond the motion of a physical robot, in the behaviour of the virtual avatar and respective feeling of force feedback. While users can manipulate the environment, we posit that the visual and haptic cues can reduce the cognitive load of interpreting the abstract concepts and make the haptic digital manipulative more expressive.

Returning to our mass-spring illustration: a physical mass connected to a real spring is a manipulative that can demonstrate the concepts of elasticity, inertia, vibrations and resonance. A programmable robot can visibly implement the mass-spring behaviour through its reactive motion. With physical user interactivity, this robot becomes a ***haptic digital manipulative***. Combined with a graphical display, it could tangibly render the system with learner-specified parameters—shape, size, spring and mass constants—and expose learners to the reaction forces and dynamics of pulling and bouncing it (Minaker et al., 2016) as well as new combinations of springs, and varying viscosity and gravitational force. Such a system can simulate many other physical systems, *e.g.*, gas, fluid or electronic circuits.

### 2.2 A Brief Overview of Haptic Force Feedback Technology Relevant to Education

Haptics is the sense of touch, and haptic technology is commonly used to refer to both tactile feedback (*e.g.*, the vibration on your smartphone) and force feedback, which acts on our proprioceptive and kinesthetic senses. Force feedback haptic devices can provide active pushing and pulling; holding the handle of one of these small robots, you can interact with a VE and the dynamics it represents. Force feedback devices come in many forms and capabilities, as portrayed on Haptipedia.org (Seifi et al., 2019).

To support the PAL vision we ultimately want a planar (2D) device that is drawing-friendly, because we see sketching experimental ideas as an intrinsic part of learning. We want large workspace to support big movements, spread out, see one’s work; and portability for working in different environments or collaborate around a table without a screen in the way. Cost is crucial to accessibility of any learning technology.

At present, these properties are in substantial conflict. In the interim, to assess technical feasibility we focused on planar (2D) world-grounded force-feedback platforms. This section describes basic terminology, intrinsic tradeoffs, and progress towards this kind of technology we need.

#### 2.2.1 Grounded Force Feedback and Impedance

Force feedback devices require a physical *ground*. Typically, *world*-grounded devices are anchored to a base in order to transfer reaction forces to a ground other than the user’s own body, generally *via* links or cables. Device *impedance* is essentially the stiffness it can display. A high-impedance device can strongly resist user movement, either by generating strong actuator forces or by braking and blocking movement. Impedance control is the most common approach to implementing force feedback models: the device is programmed to generate a force in response to a user-imposed positional input. For example, the relation *F* = *Kx* describes a simple virtual model of a spring deflection relationship.

#### 2.2.2 Workspace, Mobility and Tethering: Present and Future

With conventional GFF devices, large workspace and impedance range (the difference between minimum and maximum device-renderable impedance) are a tradeoff: 1) minimum renderable impedance *increases* with workspace due to inertia in links and actuators; and 2) maximum renderable impedance *decreases* due to longer and more compliant linkages (Barrow and Harwin, 2008; Zinn et al., 2008). Further, large devices require larger motors, stronger links, better sensors. They are heavy and expensive.

Some low-cost 2D GFF devices targeting education instead go for fabrication ease and low cost. The *Haply* (https://haply.co/) exhibits a “pantograph” configuration, which delivers consistent force over reachable workspace (Gallacher et al., 2016). The Haply has open-source construction guides and a public hAPI software library (Gallacher and Ding, 2017). We chose it for feasibility assessment because of its maturity and support.

In the future, more will be possible. To achieve mobility and large workspace, we need to consider a different approach to grounding than being bolted to a table: hand-held mobile robots which can propel themselves on a surface. Of mobile robots have been used as *active* force feedback displays, two have practical educational potential.

*Cellulo* is a mobile haptic robot purpose-designed for classroom learning, able to render virtual objects on a 2D-plane (Özgür et al., 2017a). Its omnidirectional, backdrivable mechanism uses a permanent magnet ball to generate vibration-free movement; at 168 g, the size and weight of a large pear, it costs 125 Euros. Cellulo can render variable resistive force feedback and guide users on a specified path or to a certain destination, albeit at a low sample rate and with relatively low magnitude and range of supplied force.

The *Magic Pen* is a low-cost haptic stylus (∼50$) which can provide force feedback in 2D (Kianzad and MacLean, 2018). Force grounding is supplied by friction contact between a rolling drive ball and an arbitrary 2D surface, like a tabletop or a vertical whiteboard, able to transmit resistive and guiding forces to the user’s hand. Magic Pen supports exploration of a VE, and a later version embeds the drawing capability in an assistive framework for physically assisted manual sketching (Kianzad et al., 2020). The Phasking framework introduces the concept of *control sharing* as well as *bring* and *bound* constraints as a framework to support physically assisted sketching.

Cellulo and Magic Pen are the only plausibly suitable mobile haptic displays of which we are aware which can render force feedback in the large, *i.e.*, an unrestricted workspace. The two differ in many ways: Cellulo is held in a mouse-like grip, and can move autonomously when not held; the Magic Pen is a stylus and thus more suitable for drawing applications, but cannot ambulate on its own. The Magic Pen can deliverable more controllable and larger forces, and is designed to support drawing and sketching whereas Cellulo is physically not suited for this. Purpose-designed for educational purposes, Cellulo as been evaluated in multiple learning scenarios.

### 2.3 Challenges and Opportunities in Bringing Haptics Into Educational Contexts

Several studies have explored benefits of haptics in education Magana and Balachandran, 2017; Amin et al., 2013; Jones et al., 2006).

We will next discuss the many practical issues in using haptic displays in educational settings, not least the cost of the high-end commercial haptic displays used in these studies.

#### 2.3.1 School Logistics

Besides pedagogical needs, Özgür et al., 2017a propose requirements for a useful educational platform in a class setting: it should be affordable, robust with minimum required initialization, configuration or calibration, and reliable enough to effectively support uninterrupted learning activities. Other considerations drawn from our own classroom work include extreme limits on teacher time (for studies or deployment), technological expertise, and the ability to prioritize such activities. On the technology side, it is hard to justify the expense of sole-use technology, deployment practicalities like batteries and power cords, and the sheer difficulty of students being able to determine when a device is behaving correctly. Classroom sessions are often short, requiring a quick-start system that nevertheless delivers engagement and learning gains right out of the gate (Özgür et al., 2017b).

Validation is a major challenge: it is difficult to validate learning benefit where there are countless variables and controlled studies are not possible. Thus, many studies take a qualitative approach and look for ways in which the haptic modality is changing student strategies, collaboration style, engagement and interest or type of questions (Davis et al., 2017).

#### 2.3.2 Creating Haptically Augmented Learning Environments: Open-Source Haptic Libraries

A multitude of haptic libraries support designers developing haptic VE interactions for a given technology. There are two main categories of functions: rendering haptic behaviors, and connecting the haptic interface modality to other parts of the system and experience, be it an underlying virtual model, graphics and/or sound engines and display, managing other forms of user input and control, and in some cases interaction over a network and with other users and entities. While some have been associated with a specific product, most attempt to generalize support for at least a significant class of devices (*e.g.*, CHAI3d (Conti et al., 2003), hAPI (Gallacher and Ding, 2017)).

Some of these haptic libraries support advanced rendering of complex deformation and collisions both haptically and graphically for sophisticated environments such as surgical training simulations. For educational contexts, we often do not need such complexity. For student-oriented online physics learning materials it is common to see the physical behaviour of an object presented with simplicity *via* an open body diagram and illustration of applied forces (*e.g.*, Perkins et al., 2006).

On gaming platforms, developers use graphic engines to simulate rigid body behavior in a virtual world in procedural animations which move realistically and interactively. Hapticians have exploited game engines for their VE modeling, getting graphic display for free and driving haptic output from the VE simulation; this obviates the need to make or access another physics library for haptic rendering. For example, the hAPI uses a wrapper around the 2D physics simulation library Fisica and turns it into a haptic engine system for educational purposes (Gallacher and Ding, 2017).

However, designing even a simple VE with a library requires basic knowledge of programming and physics, often absent for student or teacher, and often difficult to access in a classroom. Even when a teacher is a technology enthusiast, the uncertainty in predicting learning benefit relative to a large time investment is an understandable barrier. This underscores a broad need for more usable, accessible tools for haptic experience design which go well beyond the need for accessible technology itself.

### 2.4 Haptics for Designing and Exploring

In this survey of education-related haptics, we focus on the intersection of two primary haptic approaches: 1) haptically rendered virtual environments, and 2) pen-and-paper-based interactions. Although many devices support one or the other, only a few support both features simultaneously.

#### 2.4.1 Design Approaches: Input Methods, Feedback Modalities and CAD Features

Out of many works describing novel input and haptic output, we focus on systems suited to educational applications such as STEM learning and visual art.

*VoicePen* is a digital stylus that uses non-linguistic vocal, position and pen pressure inputs for creative drawing and object manipulation (Harada et al., 2007). VoicePen uses vowel sounds, variation of pitch, or control of loudness to generate fluid continuous input to the user’s pen interactions. *WatchPen* uses a digital stylus, smartwatch and a tablet for drawing inputs while employing vocal and touch input to reduce workflow interruptions, such as tool selection (Hung et al., 2019). These systems’ reliance on vocalization make them impractical in classrooms, but they deliver ideas for stylus interactions.

*TAKO-Pen* is a haptic pen providing pseudo-force-feedback by creating the sensation of sucking on users’ fingers through pressure chambers embedded on a handheld surface (Konyo, 2015). *RealPen* is a digital stylus which recreates the sensation of writing on real paper with a pencil through auditory and tactile feedback (Cho et al., 2016). *FlexStylus* allows users to perform tool selection and to draw accurately by bending the pen in various modes (Fellion et al., 2017). Although these novel input and feedback modalities expanded the interaction space between users and haptic devices or digital styluses, they have very specific purposes and do not point to more general sketching tools.

In addition to devices, we looked for innovations in computer-aided drawing (CAD) features for generating engineering or artistic drawings. Parametric sketching is a CAD functionality where users define geometric entities with parameters, and specify relationships between them as constraints: *e.g.*, defining a circle by its central position and radius, or defining two lines as co-linear or of equal length (Pavlidis and Van Wyk, 1985). This function is useful to architects and architects for creating complex architectural or mechanical sketches. Gürel (2019) studied the impacts of parametric drawing with CAD tools on architectural design creation, finding that allowing designers to define parameters and constraints on geometric entities enhanced creative process flexibility. Ullman et al. (1990) emphasized the importance of geometric constraint in CAD tool function for improving clarity in designers’ mechanical sketching.

For direct-sketching input, we highlight *ChalkTalk*, which recognizes users’ strokes and translates them into meaningful interactions using dynamic visualization and procedural animation to facilitate exploration and communication (Perlin et al., 2018). ChalkTalk is a purely visual medium; we see potential for using its approach when extending PAL-type functionality for more expansive sketch interpretation.

#### 2.4.2 Pen-Based Sketching Tools for Engineering Design and Educational Drawing

In pen-based devices developed for professional and education drawing, we focused on sketching on 2D surfaces to find features that suit PAL needs. In engineering design, *InSitu* provides architects with a stroke-based sketching interface capable of augmenting sites’ contextual information from sensor data into sketches and delivering the information *via* pop-ups (Paczkowski et al., 2011). *dePENd* can guide users to draw out shapes (*e.g.*, lines and circles) precisely by providing directional force feedback. It also allows users to deviate from dePENd’s guidance so she can edit the shapes at will (Yamaoka and Kakehi, 2013a).

Within educational drawing support for (STEM subjects), most devices were built for sketching math or physics diagrams and equations. *MathPad2* allows users to create animations to represent processes (*e.g.*, a mass block oscillating) in addition to static diagrams or math formula (LaViola and Zeleznik, 2004). *Hands-on Math* places more emphasis on recognizing handwritten math inputs from users and performing calculations such as solving for an unknown variable in an equation (Zeleznik et al., 2010).

While Insitu or MathPad2 support a particular type of drawing, such as architectural sketches, the PAL framework aims to support designers from a wide range of fields—architects, physicists, web developers.

### 2.5 Relevant Educational Theory and Design Guidelines

#### 2.5.1 Learning Through Experience

In Constructivism, knowledge is seen as deriving from individuals’ experiences, rather than as a transferable commodity. Learners actively construct and re-construct knowledge by interacting with the world (Piaget, 1977; Antle and Wise, 2013). According to Piaget’s cognitive development theory, to know an object means to act on it. Operation as an essence of knowledge requires the learner to modify and transform an object, and also understand the process of transformation; leading to knowledge of how the object is constructed (Piaget, 1964). Several schools of educators (Montessori and Carter, 1936; Dewey, 1938; Papert, 1980) have emphasized physicality in educational learning tools and direct manipulation of objects. These theories underlie a goal of providing tools that enable learners to operate on multiple instances of knowledge construction.

#### 2.5.2 Extending Experience With Reflection

Meanwhile, Le Cornu (2009) propose three iterative steps of *externalization*, *sense-making of meaning*, and *internalization*, through which reflection links experience to learning. Often discussed in social constructionism literature, these steps have been applied to a wide range of human actions in the world and society, including the use of feedback (from people, or the results of physical “experiments”) to develop the meaning of the self.

#### 2.5.3 Haptic Digital Manipulatives as Vehicles for Experience and Reflection

The theories above have been applied to a wide range of tangible user interfaces and digital manipulatives. Through educational robots, experiential learning can be tangible and digitally supported, and specifically invite reflection. Resnick et al., 1998’s process of *reflection with robots* starts with the construction of a robot-based environment, in which learners make their own “microworld” by programming it, followed by feedback from robots to help them shape and validate their ideas. Such a reflection cycle can be repeated multiple times, deepening the experience (Frei et al., 2000).

Within early edurobot work, we sought visions for digital manipulatives suitable for more advanced educational topics. We found examples using robots to aid learners in mindful integration or materialization of ideas through the practice of design (Ackermann, 2020); and to support exploration of different domains of knowledge or of abstract concepts by making them more accessible or approachable in new ways (Özgür et al., 2017c).

Instantiating these principles in a digital manipulative could help them to work as an *object-to-think-with*, wherein learners instantiate their ideas into a physical model through the object, and can debug or extend their thinking model regarding the outcome. The process of analyzing the validity of execution motivates learners to think about their own thinking, developing their metacognitive ability. This results in 1) gaining higher-level thinking skills, 2) generating more representations and metaphors for their understanding, 3) improving social communication and a collaborative atmosphere, and 4) forming deeper understanding of the concept among learners (Atmatzidou et al., 2018; Blanchard et al., 2010).

## 3 A Framework for Physically Assisted Learning

The motivation for the PAL framework is to exploit benefits postulated above for a haptic digital manipulative, in learning and in pen-and-paper interaction, and turn them into a versatile and effective digital manipulative. We previously introduced Kolb (1984)’s four-stage framework for experiential learning, on which we have based PAL (Figure 1). Here, we lay out PAL’s theoretical basis, then elaborate on its components and explain how we expect learners and designers to use it.

### 3.1 Pedagogical Rationale and Components

Learning is iterative: one builds a mental model of a concept by repeatedly interrogating and manipulating a system, forming then testing successive ideas of how it works in a cycle such as Kolb’s. Manipulatives are often designed in a way that will support just one part of this cycle—*e.g.*, to create a microworld *or* to directly interact with one.

Our premise is that supporting fluid movement *throughout* the experiential learning cycle will facilitate more resilient mental model formation.

#### 3.1.1 Supporting Kolb’s Learning Stages With a Haptic Digital Manipluative

Most of the visions in Section 2.4, and the idea of robot-supported reflection more broadly, would support at least one out of Kolb’s two “acting in the world” phases: Concrete Experience (CE; having an experience) and Active Experimentation (AE; putting a theory into practice). Here, there is an opportunity for intervention, and also for researchers to observe and try to understand what is happening based on the part of the cycle that is visible. The more internal stages of Reflective Observation (RO; reflecting on an experience) and Abstract Conceptualization (AC; theorizing) are crucial, but can be influenced or inferred only through what happens in the other phases, or through post-hoc assessment, *e.g.*, of changes in conceptual understanding.

The PAL framework’s mandate is therefore to help educators focus on physical instruments and strategies that will support learners in CE and AE, and eventually to help us insightfully observe them as they do so.

Early works on edurobots have claimed that robots could be beneficial in all four stages. For example, for Reflective Observation (RO), Resnick et al., 1998 suggested that through its processing power, the robot could speed the reflection cycle—externalizing/internalizing from hypothesis to result; modifying parameters, conditions and even time. For Abstract Conceptualization (AC), Papert (1980) uses gears as an example where learners can use mechanical objects for conceptualizing physics concepts.

Kolb himself argues that the *interaction and manipulation* of tangible objects is an indivisible part of epistemic (knowledge-seeking) exploration, where the learner purposefully changes the learning environment to see its effect and thereby to understand relationships. When suitably framed through availability of multiple perspectives, parameters and factors, manipulation thus might provide at least indirect support for Kolb’s Reflecting Observation (RO) stage (Antle and Wise, 2013; Fan et al., 2016).

However, these claims are as yet unsupported. Limited to findings that have been validated in controlled studies, we conjecture that a DM approach’s influence on RO and AC will be indirect.

#### 3.1.2 Physically Assisted Learning Components

A useful (that is, versatile) manipulative should be able to provide the basis for productive subsequent reflection and theorizing during both Active Experimentation (AE) and Concrete Experience (CE). Therefore, **we identified *explore* (CE) and *design* (AE) as PAL’s key components: activities which a haptic DM must enrich**.

Further support for centering a framework on these two components, as well as clues towards means of implementing them, emerge from other studies of how haptic feedback can support *designing* and *exploring*. Summarizing these, Table 1 has two features of particular interest. First, we populated it with just two of Kolb’s four learning activities, because we found very few examples of attempts to use haptics or other PDMs to directly support reflection or theorizing. Those we did find (*e.g.*, Hallman et al., 2009; Reiner, 2009; Triana et al., 2017) proposed systems or studies whose results either showed no benefit or were inconclusive.

**TABLE 1:** Summary of research informing the use and benefits of haptics in learning, organized by the PAL framework’s two activity components. [+] indicates a positive benefit, or [−] no added value was found.

Haptic benefits	Design (Active exploration)	Explore (concrete experience)
Understanding and manipulating geometry	[+] Yamaoka and Kakehi (2013b) Drawing accurate geometric shapes	[+] Özgür et al. (2017c) Identifying different shapes and number of edges
	[+] Nakagaki and Kakehi (2014) Computer assist collaborative drawing of different shapes	[+] Minogue and Jones (2009) Understanding the structure and function of the cell membrane transform
	[+] Lin et al. (2016) Increasing the passive stylus affordance through haptic guidance	[+] Jones et al. (2006) Learning morphology and dimensionality of viruses; diagnose mysterious viruses by pushing, cutting and poking
Improving accuracy and speed	[+] Kianzad et al. (2020) Improving accuracy of drawing objects through force feedback assistance	[+] Murayama et al. (2004) Enhancing completion time and interactivity of bimanual tasks
	[+] Wang et al. (2006) Using haptic feedback in a calligraphy simulation reduces writing errors and improves writing speed	[−] Evans (2005) Users were unable to sculpt forms to produce acceptable curved surfaces using haptic feedback
	[+] Yamaoka and Kakehi (2013b) Drawing accurate geometric shapes	[−] Beckers et al. (2020) Haptic human–human interaction does not improve individual visuomotor adaptation
Engagement	[+] Hamza-Lup and Stanescu (2010) Significant increase in students’ engagement during the learning activity	[+] Vaquero-Melchor and Bernardos (2019) Enhancing interactions with objects in Augmented Reality
	[+] Younq-Seok et al. (2013) Increasing engagement in word-writing activities	[+] Tsetserukou et al. (2010) Providing realistic sensation of physical interaction in a virtual environment
	[+] Kyung et al. (2007) Increasing confidence and achieving more realistic drawings	[+] Khodr et al. (2020) More engagement in educational robotic activities
Accessibility (e.g., in face of disability)	[+] Mullins et al. (2005) Re-learning to write after a stroke	[+] Wall and Brewster (2003) Allowing visually impaired users to perceive data with greater speed and efficiency
	[+] Jafari et al. (2016) Haptics improves task performance of children with physical disabilities (review paper	
Understanding of underlying concepts	[+] Lopes et al. (2016) Designing an optimum system/model by receiving on-the-go force feedback	[+] Magana and Balachandran (2017) Conceptualizing electrostatic concepts through the sense of touch
		[+] Zacharia and Michael (2016) Building electrical circuits with one or two bulbs
		[−] Renken and Nunez (2013) Haptics did not add to learners’ ability to understand pendulum principles
		[+] Zacharia et al. (2012) Understanding mass-beam balance

Secondly, none of the cited studies examined *both* designing and exploring, but treated them as isolated activities. This may have been influenced by the natural affordances of the devices used. For instance, a Haply (in its unmodified state) can be used readily to *Explore*; but to facilitate creation of micro-worlds (*Design*), we felt we needed to hack it—and chose addition of a drawing utensil. In other words, meeting the principles expressed by PAL triggered specific, targeted technology innovation. More is needed to reach the full PAL vision; the framework provides a blueprint to get there.

### 3.2 Principles for Creating Digital Manipulatives

We assert two overriding principles that guide us in creating versatile digital manipulatives, based on learning theory discussed in Section 2.5 as well as observations of learners’ interactions both with conventional pen and paper and with haptic/robotic devices, across a range of learning scenarios.

#### 3.2.1 A Digital Manipulative Needs to Serve Learners in Expressing Their thoughts (Design)

According to Ackermann (2020), “*To design is to give form or expression, to inner feelings and ideas, thus projecting them outwards and making them tangible*”. Design enables individual interactions with and through human made artifacts and involves them in the “world-making” process (Goodman, 1978). The purpose of design goes beyond representing just what exists, by bringing imagination into this existence (Ackermann, 2020).

For example, we often use pen and paper to write down fast-travelling ideas in our minds. Our immediate drawings can reflect our thoughts, experiences and emotions. Particularly for children, drawings reveal the hidden transcripts of their interpretation of the world.

From scribbles to detailed, elaborated productions, sketching is both intellectual play and can help us form, develop and communicate our thoughts, a key part of a conceptual process. Sketching is direct, improvisational, expressive, resists distraction, and may promote deeper cognitive processing. Projecting our ideas onto paper makes our thoughts more tangible, shareable, and justifiable; This enhances our communications with others. A versatile manipulative should work as a medium to exchange information between a user and a computer interactively.

These prior findings and observations support the premise that aid from a suitably configured and supported physical digital manipulative can directly impact the active experimentation phase: specifically, when learners are hypothesizing and planning small tests. The environment altogether should encourage the learner to hypothesize, construct a experimental micro-world and set the conditions for the environment, anticipate the result and test it; and iterate to improve their hypothesis.

#### 3.2.2 A Digital Manipulative Needs to Support Exploration of Domains of Knowledge (Explore)

Two classes of manipulative proposed by Resnick et al. (1998) include *Frobel* Manipulatives (FiMs) to model the world, *i.e.*, provide an intuitive way to experience many concepts in physics by making them more accessible (wooden sphere and cube to feel the natural differences between shapes), and *Montessori* manipulative (MiMs) to model abstract structure—*e.g.*, form an approachable way to make math, and geometry concepts more tangible (golden bead materials used for representing number). Haptics researchers show that even a 1D haptic device can support both of these classes when it works as haptic mirror (Minaker et al., 2016), to mimic physical experience, or as a haptic bridge, connecting a dynamic visualization of a mathematical concept with a haptic representation (Davis et al., 2017). A versatile manipulative should support both classes using physical interaction with the virtual world through force feedback.

Perhaps the most studied aspect of digital and physical manipulative is the role of physicality in simulation learning for concrete experience (CE) stage. Here, learners try out the action and have a new experience. Through physicality, learners can obtain more embodied experiences and perceive information through touch.

### 3.3 Using the Physically Assisted Learning Framework

#### 3.3.1 Learner’s Use

Some examples illustrate PAL’s two conceptual activities, wherein a learner constructs a microworld then explores it.

*Design:* The learner must be able to fluidly express rich information to the system. Assistive force feedback to users’ pens while sketching can help them manifest and communicate their ideas to other people and to a computer: it might be more efficient and natural if they can feel virtual constraints that support them in generating smooth curves and straight lines as they draw—on a computer screen, paper, whiteboard or other surface. In the future, we can exploit this design space to empower learners to actively design, make, and change their learning environment based on their hypothesis.

*Explore:* The tool must provide rich sensory information to the learner. The addition of haptics to a digital manipulative (beyond motion alone) potentially supports a more compelling interpretation so that learners can predict and reason about outcomes based on what they feel as well as see.

In this project we explore these two PAL activities—requisite attributes for an object to think with—along with the connection between them. Although such a device could also be seen as an object to promote computational thinking (Ioannou and Makridou, 2018) we saw it differently. A DM exploits the computational power of the computer to speed up the *learner’s* reflection cycle, which leads to more constructive failures (Clifford, 1984). Throughout this process, learners can explore a variety of representations and solution methods. If followed by a consolidation and knowledge assembly stage, together they can create a productive failure process (Kapur and Bielaczyc, 2012).

#### 3.3.2 Education Technology Designer’s Use

##### 3.3.2.1 Ideation of Form and Prediction of Haptic Value

Designing technology solutions for learning requires ideating innovative concepts and ideas, but also evaluating and prioritizing them. PAL can help inspire educational technology designers with new ideas, and to understand the potential of adding haptics to a particular domain or context. In addition, our implementation shows a technical example of how to use emerging technological capabilities to solve particular problems.

##### 3.3.2.2 Setting Requirements and Evaluating the Result

PAL can help designers identify *requirements via* experiences that their technology needs to support. Based on Figure 1, a designer can create an opportunity map by examining connections between the stages of learning and activity type.

For example, to support collaboration in learning electrostatic forces, a learner can construct the environment (design) by placing the point charges; then invite their partner to experience them (explore). A designer can then focus on finding the haptic controls and feedback which will allow the learner to place the point charges correct places (*e.g.*, equidistant), and how to render the force behaviour as learners move respectively to each other.

Based on these requirements, in *evaluation* a ed-tech designer simply needs (at a first pass) to verify that the requirements are being met when learners interact with the system. Are they able to construct the environment, and then place the charges correct? Can a partner experience this? Is the whole experience engaging and usable enough to invite this kind of collaboration? With the assurance provided by intermediate goal and usability evaluation derived from theory-based guidelines, they will be in a better position to proceed to assess how such a system is influencing learning outcome.

### 3.4 First Step: Need for a Technical Proof-of-Concept

In past research supporting haptic *design* and *explore* activities (Table 1), what is missing is the *connection between* them.

This requires a technical means by which to understand the user’s imagination and dialogue in *design* and then bring it into existence by defining its physical, haptic behaviour for *exploring*. For example, if a user draws a microworld consisting of a set of point charges, we need to define the force behaviour of the point charge and make it interactive so that users can feel the forces as they move in the environment.

Once such a system exists, it can misfire for purely technical reasons. For example, expanding the user’s available possibilities during *design*—*e.g.*, allowing them to cover a greater variety of concepts in more ways—often introduces new issues such as triggering vibrational instabilities which naturally accompany haptic rendering of dynamic environments with large uncertainties.

In summary, the challenges here are to 1) make an intelligent system that can take unconstrained drawing as an input, and 2) robustly render a wide range of haptic environments with high quality. For the first, advances in artificial intelligence go far in allowing us to infer and display interpretations of user’s drawings (Bhunia et al., 2020; Dey et al., 2019). For the second, the field of haptic rendering can contribute advanced control methods which when carefully applied should be able to describe and within bounds, to address the environments that may arise when a user is permitted to create ad hoc environments Haddadi et al., 2015; Diolaiti et al., 2006.

Putting these elements together is, however, a substantial systems-type contribution, and its initial appropriate validation is in technical performance assessment with respect to force and stability outputs relative to known human psychometric capabilities rather than a user study of either usability or learning efficacy. In the following, we will describe and assess performance of our technical-proof-of-concept system which implements this missing, connective aspect of our proposed PAL framework.

## 4 Haptically Linking the Expression and Exploration of an Idea

Currently available processes for generating and modifying content for haptic interaction (Section 2.3) impose logistic and cognitive friction between ideation in the form of sketching a problem, idea or experiment the learner would like to understand, and testing that idea in the form of a physicalized model. We aim to reduce this friction.

**FIGURE 2:**
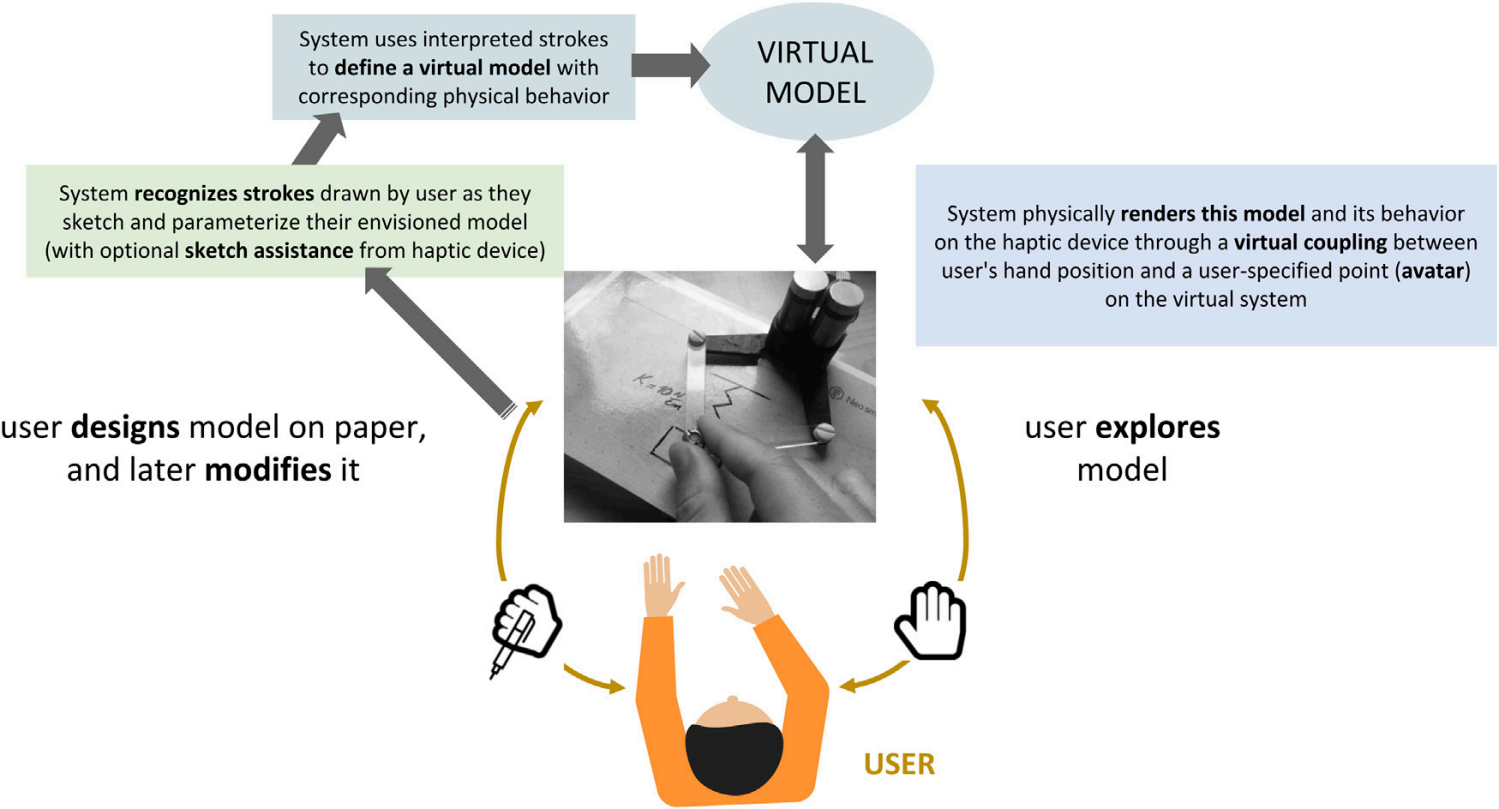
Technical implementation required to support *Design* (green) and *Explore* (blue) learning activities in response to ongoing user input. Details are explained in Section 4.3. (The user’s graphic from Can Stock Photo, with permission).

After describing the technical setup we will use to demonstrate our ideas (overviewed in Figure 2), we will work through a series of technical instantiations which support increasingly powerful and wide-ranging cases. Each begins with an education use case illustrating how this level of haptics could be useful. Readers may find the first (rendering a haptic wall) distant from our final goal; we have included it as a means of gradually exposing layers of haptic technology needed to understand more complex implementations. While all of the haptic rendering algorithms described here are well known, we show how they can be combined in new ways with other technical features (*e.g.*, stroke recognition) to meet technical challenges that arise from the requirements of a versatile, unrestricted learning environment.

### 4.1 Technical Proof-of-Concept Platform: Haply Display and Digital-Pen Stroke Capture

**FIGURE 3:**
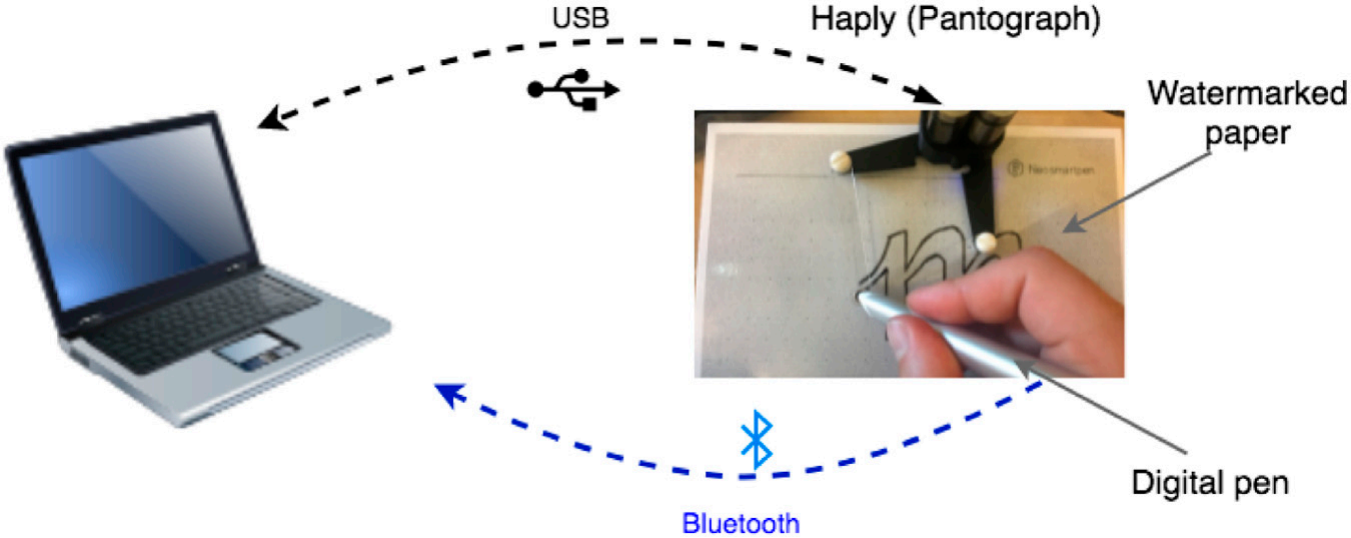
Technical Setup. Our demonstration platform consists of a Haply force-feedback pantograph, a USB-connected digital pen, and a host computer. The Haply communicates position information to the host computer and receives motor commands through a USB port. A digital pen captures and conveys thes user’s stroke, along with data opressure, twist, tilt and yaw.

The demonstrations described here use the Haply Robotics’ pantograph system (Figure 3, https://haply.co/, Gallacher and Ding, 2018) and its hAPI software library (Gallacher and Ding, 2017). The Haply is a low-cost pantograph, relying on 3D-printed parts which together with good-quality motors and fast communication can offer convincing haptic rendering with respect to accuracy, force levels, responsiveness and uniformity across its 14 × 10 cm workspace (https://haptipedia.org/?device=Haply2DOF2016). It communicates sensor and actuator data *via* USB to a VE running on a host computer, typically using the Processing computer language. The hAPI library renders haptic interactions by reading the pantograph’s end-effector position (moved by the user’s hand) and computing output forces sent to two driver motors.

To capture users’ sketch strokes, we used a watermarked paper and a digital pen (Neo Smart Pen, Inc. (2018)) connected to the Haply end-effector. The digital pen captures detailed information about the user’s stroke: absolute position, pressure, twist, tilt and yaw. The Neo pen requires watermarked paper, creatable with a standard laserprinter by printing encoded dot files. For erasability and re-usability of sheets, we laminated the watermarked paper and positioned it under the Haply workspace. We calibrated the digital pen’s position data with the Haply’s encoders. With this system, the user can draw on the laminated paper and the strokes are captured, sent to the host computer and imported to the Processing application that interacts with the Haply.

### 4.2 Level 1: Rendering Rigid Surfaces and Tunnels

We begin by illustrating how haptics could potentially support learning (in motor coordination) with basic haptic rendering techniques.

#### 4.2.1 Use Case: Handwriting Training Guided by Virtual Walls

Past research on motor training, *e.g.*, post-injury rehabilitation, has elucidated effective strategies for utilizing physical guidance, whether from a human trainer or a programmed haptic appliance. Full guidance of a desired movement does not typically produce good transfer to the unguided case; some studies suggest better results by physically obstructing the desired movement in the face of visual feedback, causing exaggerated motor unit recruitment (Huegel and O’Malley, 2010; Fu et al., 2014). Learning and improving handwriting similarly involves training numerous haptic sensorimotor activities; these employ both fine (fingers) and larger (arms) motor units. It entails significant mental and motor-control practice, particularly for individuals working against special challenges, such as dysgraphia which can impact 25% of the school-aged population (Smits-Engelsman et al., 2001; Guneysu Ozgur et al., 2020).

However, learning and improving handwriting is also a cognitive practice, and often practiced by the young where engagement is also important. Rather than learners comparing their results to a standardized specified outcome, an expert may be able to conceive of better individualized support (more specific, or advanced at a different rate) but requires a means to convey it to the learner as they practice on their own (Gargot et al., 2021).

The priority may thus be easing an expert’s customization of exercises, to support repeated self-managed practice (Guneysu Ozgur et al., 2020). The expert might want to modify details of visual cue presentation and the level and form of haptic guidance (Korres and Eid, 2020; Teranishi et al., 2018); or temporally adapt by reducing force feedback aid over time through control-sharing (Kianzad et al., 2020). Effective feedback must convey correct movements, notify a learner when something goes wrong, and show them how to correct their movement (Asselborn et al., 2018; Amin et al., 2013). Haptic guidance could potentially provide these needed cues when the teacher is not present, without demanding a high cognitive load.

In the PAL framework, the teacher would use the *design* stage, then *explore* to ensure the force feedback works correctly. The learner would access this resource in the *explore* stage.

Here, we show in a basic example targeting elementary school students how a teacher can define a channel within which the learner needs to stay as they trace a letter. This channel will be rendered as a pair of enclosing and guiding haptic walls. This simple demonstration does not attempt best practices for handwriting training, or demonstrate many customization possibilities; it primarily introduces a important building block of haptic rendering, but is also a placeholder for the advanced ways listed above that haptic feedback could be used ti customize handwriting support.

#### 4.2.2 Defining a Wall

There are many ways to define a boundary to a computer program. We require a means that is convenient for a teacher or therapist. Working in a context of pen-and-paper, we let the teacher sketch the path which they wish the learner to follow. Their strokes are captured as a time-based set of point coordinates. These can be used either directly, if the stroke sample density is adequate, or with a smoothed line fit to them.

We collect the user’s strokes as a two-dimensional array, then re-sample it with spatial uniformity and present the result as a one-sided wall. A user can move freely on one side of the wall; if they penetrate the wall from the free direction, they will feel resistance. A teacher can draw a set of one-sided walls as a letter-shaped tunnel to guide a learner in their handwriting practice.

#### 4.2.3 Feeling the Wall: Virtual Coupling

**FIGURE 4:**
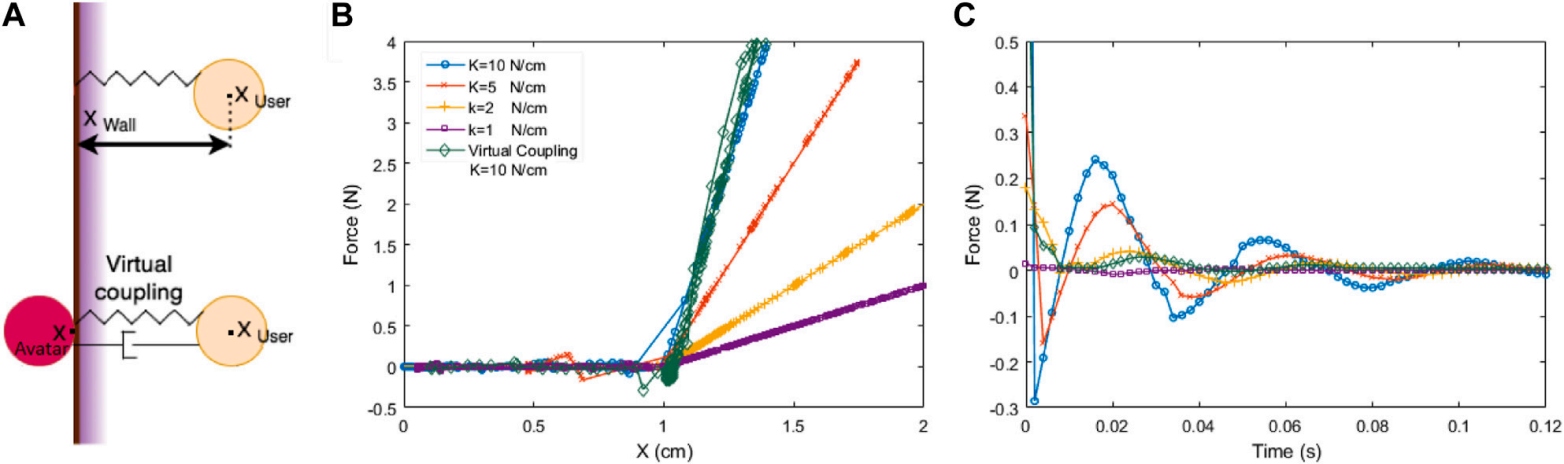
Rendering a haptic wall, using a virtual coupling to achieve both high stiffness and stability. **(A)** Algorithm schematic. **(Upper)** In the simplest rendering method, force depends directly on the distance between the virtual wall and the user’s hand (haptic device) as it penetrates the wall: *F* = *K* (*X*_*wall*_–*X*_*user*_). **(Lower)** A *virtual coupling* establishes an *avatar* where *X*_*user*_ would be if we could render a wall of infinite stiffness, and imposes a virtual damped-spring connection between *X*_*user*_ and *X*_*avatar*_. **(B)** Force-displacement behaviour when the wall is rendered as a direct stiffness or through a virtual coupling. The VC used here also uses the maximum *K* = 10 *N*/*cm*, and achieves a similar stiffness as when this *K* value is used on its own. **(C)** Oscillatory behavior of the conditions from **(B)**. In direct rendering, instability increases with *K*, but with a VC, a high *K* is as stable as the softest direct-rendered wall. **(B)** and **(C)** show data sampled from a Haply device.

The simplest way to haptically render a wall is to sense the position of the user or haptic device handle, hereafter *X*_*user*_, and compare it with the wall boundary *X*_*wall*_. If *X*_*user*_ has penetrated *X*_*wall*_, the penetration distance is multiplied by a stiffness *K* defining the force that pushes the user out of the wall (Figure 4A, upper). However, we typically want to render very stiff walls, while limitations of haptic device force output and sampling rate create a result which is both squishy and unstable (Gillespie and Cutkosky, 1996). As shown in Figure 4B,C, increasing *K* makes a more rigid wall but at the cost of unstable oscillations.

#### 4.2.4 Virtual Coupling for Stiff Yet Stable Walls

An accepted technique for stably rendering stiff walls, *virtual coupling* connects the haptic end-effector position *X*_*user*_ to a point representing it in the virtual world which we define as its **avatar** (*X*_*avatar*_, Salisbury and Srinivasan, 1997). A VC links *X*_*user*_ to *X*_*avatar*_ through a virtual damped-spring, as shown in Figure 4A, lower). A stiff VC spring connects the operator more tightly to the virtual model; they can feel more detail, but it can lead to instabilities.

Thus, a VC’s parameters (stiffness and damping) need to be tuned to model properties, such as virtual mass and spring magnitudes, device force limits and anticipated interaction velocities. When these are known and constrained to a limited range, a VC can work very well. The VC implementation in the hAPI interface library enables users to change VC parameters (Gallacher and Ding, 2017).

A virtual coupling is closely related to a proportional-derivitive (PD) controller, perhaps the most basic form of automatic control structure. The key goals in tuning either system are to 1) set damping to the minimum needed for stability, to limit energy dissipation and consequently responsiveness; balanced with 2) sufficient stiffness to achieve satisfactorily tight connection to the user’s motion. System stability is also challenged when the mass of the virtual entity to which the avatar is either bound or touching is too small, or when the system’s update (sampling) rate is slow compared to the dynamics of the system (either the virtual system or the user’s movement) (Shannon, 1949).

#### 4.2.5 Wall Performance in Letter-Drawing Use Case

**FIGURE 5:**
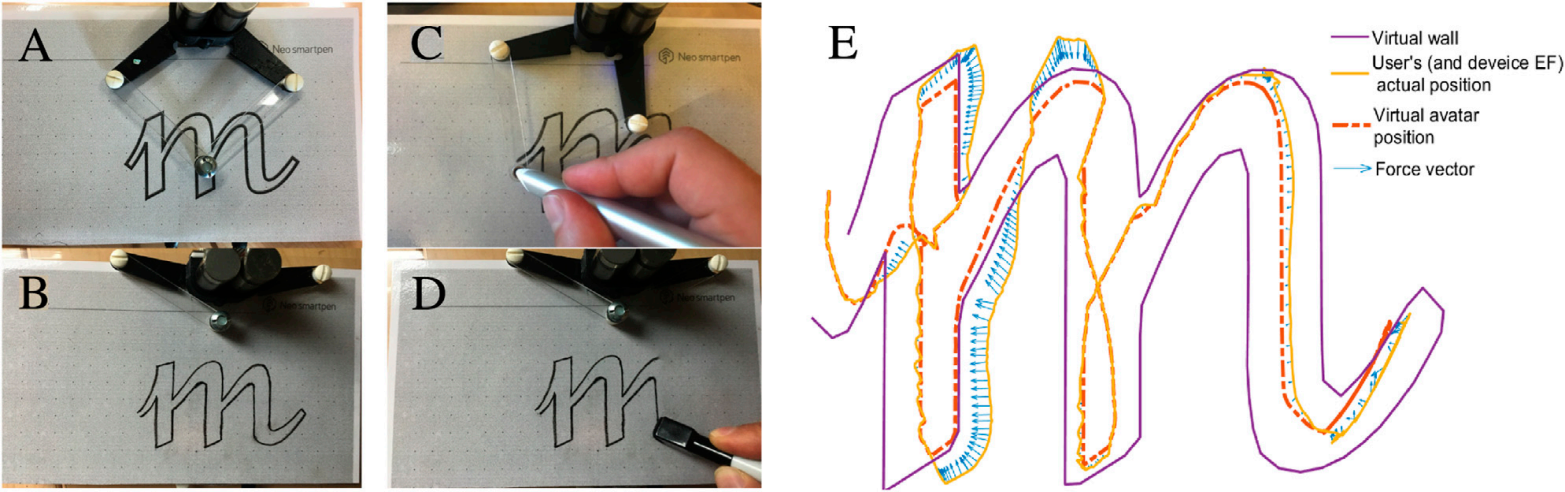
A teacher prepares a handwriting activity by defining a letter shape *m*; the learner will then attempt to form the letter with assisting guidance. To create the *m*, the teacher can **(A)** laser-print a computer-generated graphic on paper, **(B)** draw it by hand, or **(C)** manually draw it with haptic assistance. For erasable media, *e.g.*, pencil on paper or marker on whiteboard, the teacher can **(D)** erase and draw a new exercise. **(E)** Exploring the *m* with ink marks rendered as virtual walls.

In Figure 5, we show the various mechanisms by which a teacher can define and revise a shape which they want a learner to trace (A–D). In (E), we show an example of a learner *exploring* the tunnel defined by the letter outline, including the haptic rendering performance of the virtual coupling as a learner practice to write an *m*. The spring-damper VC filters high frequency force variations and creates smooth guidance as the user slides between and long the walls; the forces keep them within the tunnel. The user’s actual position sometimes goes outside the wall, but their avatar remains within it and the learner feels restoring forces pulling them back inside. Depending on velocity, the user position and avatar may be slightly displaced even while within the wall, as the user “pulls” the avatar along through the damped-spring coupling.

To extend this example, a teacher could adjust the tunnel width (a step amenable to parameterization) to customize the experience for the learner. The activity can optionally be visualized graphically, or be done entirely on paper. Learner progress can be quantified through statistics of position error (distance between the physical and virtual avatars) and the force magnitude generated in response to this error.

### 4.3 Level 2: Drawing and Feeling Dynamic Systems

Our second example implements more challenging stroke recognition, and addresses the situation where a virtual coupling is inadequate because of the range of properties that the user may need to access in their design and exploration. The overall flow of the interaction is illustrated in Figure 2.

#### 4.3.1 Use Case: a Mass-Spring System

**FIGURE 6:**
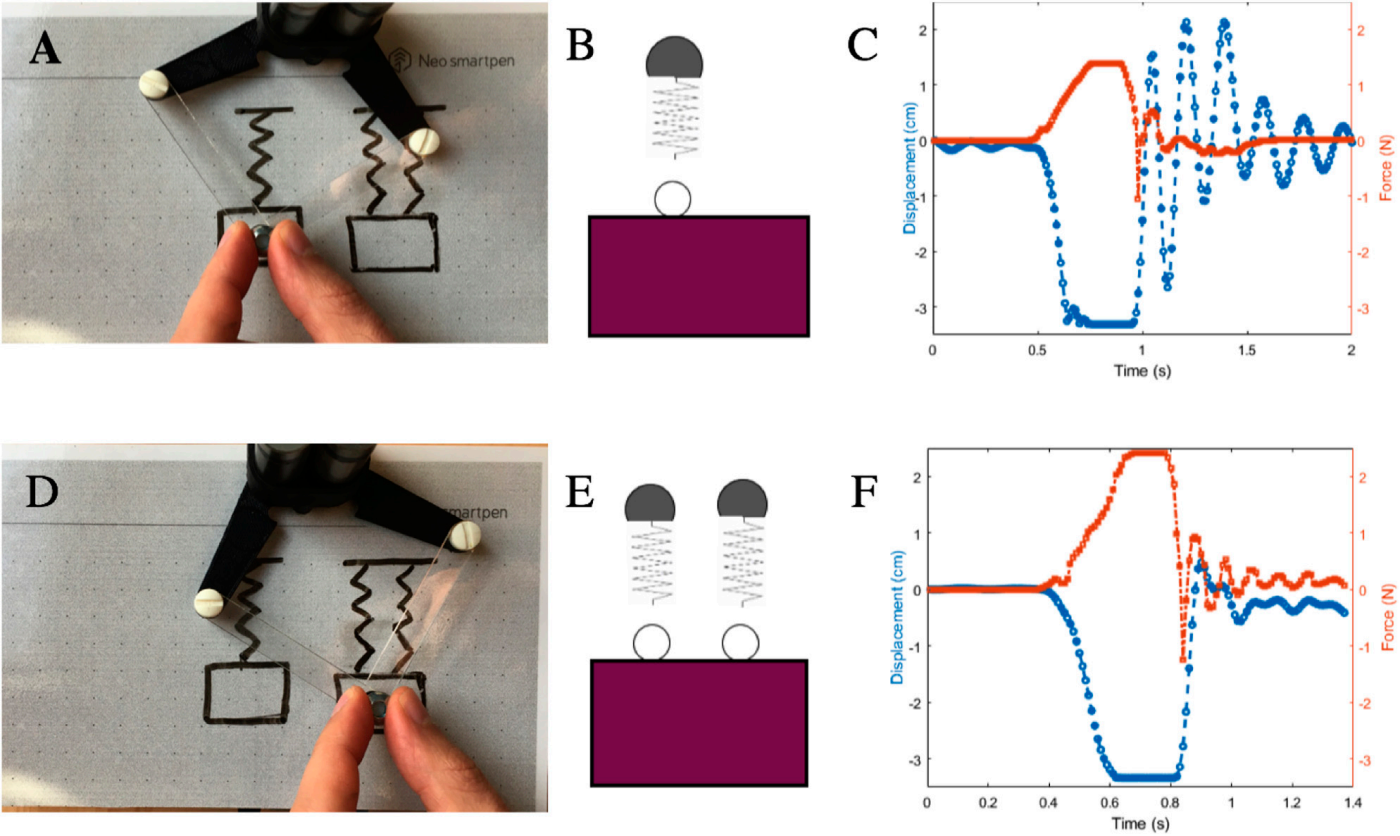
Use case: comparing the dynamic behavior of different spring–mass system configurations by drawing then feeling. **(A)** The user sketches a pair of spring–mass systems using a system-readable notation. **(B,E)** Our system recognizes the user’s strokes and incorporates them into virtual models. The user can now “connect” to one of the drawn masses by moving over it and *e.g.*, clicking a user interface button. **(C)** Behavior when connected to the single-spring configuration **(A)**. The system implements the corresponding model **(B)** by pinning *X*_*avatar*_ to that mass. The user can then feel the oscillatory force behaviour by “pulling the mass down”, extending and releasing the spring. **(D)** The user connects to the two-parallel-springs configuration, and compares its behavior (model E) to the first one. **(F)** compared to **(C)** shows a higher force for the same displacement, and a different oscillatory behavior. This system is implemented using a passivity controller to allow a wide range of M and K values, which are modifiable by hand-writing new values on the sketch.

Hook’s Law is a linchpin topic in high school physics: along with gravity and friction, students learn about the relation between applied force and the amount of displacement in springs and other stretchable materials. They further must be able to define what a spring constant is, how to compute a net constant assembled through parallel and serial spring assemblies, and with support from their teacher, conduct experiments to verify spring-stiffness hypotheses (Giancoli, 2005). Here, we use a dynamic system consisting of coupled mass and springs to demonstrate the construction of and interaction with a physical system model based on the PAL framework (Figure 6).

#### 4.3.2 System Interprets the User’s Stroke

We used a 2D recognition library implemented in Processing (the *$1 Unistroke Recognizer* (Wobbrock et al., 2007) to translate user sketches into a virtual model. *$1* is an instance-based nearest-neighbor classifier with a 2-D Euclidean distance function. It can accurately identify 16 simple gesture types, *e.g.*, zigzag, circle, rectangle. To improve performance and customize it to shapes relevant to models our system supports, we created a database to which learners can add their own labeled strokes. In the current implementation, the system starts in a training mode where users draw then type to label their sample; then exit training mode and start designing their experiment.

Our current implementation is modal: it needs to know what kind of a system a user is sketching in order to recognize their marks. A zig-zag could represent a spring in a mechanical system, or a resistor in an electrical circuit. This can be done by manually writing the system type’s name on the paper with the digital pen as shown by Lopes et al., 2016—*e.g.*, “Hydraulic lab” triggers a hydraulic simulation. The Tesseract optical character recognizer (OCR) system is one of many robust solutions (Kay, 2007). For simplicity, we selected environments using a graphical user interface.

Reliance on a set notation for sketching has a potential as usability feature or pitfall. If the notation is well known (*e.g.*, taught in the curriculum), it gives the learner a pre-existing language; versus unfamiliar, unmemorable or uncued (*e.g.*, no “tool-tips”). We did not focus on usability refinement at this stage; ensuring it will be an important future step.

#### 4.3.3 System Interprets User Strokes for Model Construction and Parameter Assignment

Ease of environment specification and modification is an important PAL principle. One way that users can specify environment parameters is in the way they draw them. For a mechanical system, a box indicates a mass; mass magnitude is interpreted as the area within the box. Spring stiffness is assigned based on the zigzag’s aspect ratio. Haptics can provide assistive guidance to create more accurate drawings. Here, haptic constraints help the user follow implicit geometrical relationships such as relative locations and sizes, through “snapping”; thus the user can perceive when they reach and move beyond the width or length of the previously drawn spring.

Some parameters are harder to indicate graphically, or the user may want to modify an initial value. This could be handled by writing an equation: *e.g.*, set the value of gravitational force with *g* = 9.8 m/*s* ^2^, or change a spring constant by *K*_1_ = 10 *N*/*cm*. As before, recognition can be done with an OCR like Tesseract, a possibility already demonstrated by at least one other system (Lopes et al., 2016).

#### 4.3.4 Unconstrained Experimentation Requires Stepping Up the Control Law

Fluid exploration means that a learner should be able to observe and feel an object’s behaviour and reason about it. This requires changing object properties, comparing behaviour between versions of a model and reflecting on the differences.

Above, we introduced the concept of an avatar as key to rendering a wall through a virtual coupling. The avatar’s existence was transparent to the user, its existence implicit in their movement. But when we advance to interacting with multiple dynamic systems—to compare them—users must get more explicit with their avatar. To “hold on” to and interact with a part of a virtual model, such as a tool or to probe part of a dynamic system, they must hitch or pin their avatar to that model element, just as they might when selecting a character in a virtual game.

The combined functionality of 1) pinning and unpinning one’s avatar to arbitrary system elements, and 2) allowing unconstrained parameter assignment, is a major departure from how a model intended for haptic rendering is typically constructed. Normally, we design an environment with particular components, set its parameters to a pre-known range, and expect the user to interact with it in a particular set of ways—always connecting through a particular avatar linkage. For example, in a surgical simulation, we might have a defined set of tools, and known tissue parameters. Bone and liver have very different properties, and rendering them might be highly complex and computationally expensive, but their properties are known in advance. We can tune a controller (such as a VC) to work with those constrained conditions.

This is no longer the case if parameters can be changed arbitrarily and on the fly, and as usual, the result will be instability. Commonly, several factors can cause instability, such as quantization, delays, and virtual object properties like stiffness and mass. We address this next with the passivity controller.

### 4.4 Level 3: Expanding the Range of Parameter Exploration Through Passivity Control

To move beyond the simple tuning heuristics above, we reference the notion of *passivity*. A real-world, nonvirtual system like a wood tabletop or mechanical button or doorknob is *energetically passive*—it will never vibrate unstably when we touch, tap or wiggle it because such oscillations require additional energy which they cannot access. The only energy flowing into the interaction comes from our own hand. At best, we can excite a mechanical resonance (*e.g.*, by bouncing a rubber ball, or pumping our legs on a swingset), but this cannot grow in an unlimited way because of the lack of an external energy source.

In contrast, a haptic display is *energetically active:* it accesses an external energy source through its controller. This is necessary for the system to physically simulate a VE’s dynamics. However, instability—often manifested as vibrations that grow without bounds, or unnaturally “buzz” upon operator contact—occur when the total energy entering the system from the human operator and the controller’s commands is greater than the energy leaving it.

*Passivity theory* underlies a type of controller which can be designed so as to guarantee stability in systems interacting with humans (Colgate and Brown, 1994; Colgate and Schenkel, 1997; Miller et al., 2004). In essence, passivity controllers bound system movements based on the flow of energy flow through the system: they guarantee overall system passivity by ensuring that the energy input exceeds outputs. It also can achieve global stability through local passivity in subsystems separately. As a result, if we know that other parts of the virtual model and physical device are operating in a passive range, we can focus on the subsystem that the (less predictable) user is interacting with.

#### 4.4.1 Passivity Controller Overview and Design

**FIGURE 7:**
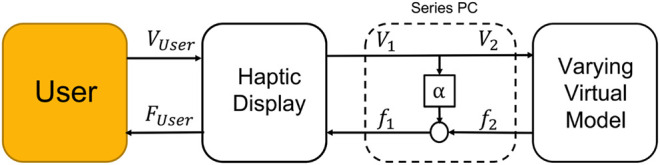
Simulation model of a complete haptic interface system and passivity controller, as implemented here. [Reproduced from Hannaford and Ryu (2002), Figure 8—*permission not obtained for review manuscript*]. System blocks are (left to right): user, haptic display, passivity controller α, and virtual environment.

We designed our *passivity controller* (PC) with the method described by Hannaford and Ryu (2002). In overview (Figure 7), the PC is interposed in series between the haptic interface and VE. This location is similar to the virtual coupling controller, and like the VC, the PC works by acting as a virtual dissipative element; the PC differs from a VC through its more targeted energetic accounting system.

The human operator interacts physically with the haptic device in continuous time; however, since the control system is digitally sampled, the VE is computed with a time delay typically specified at 1/10 of the fastest dynamics in the system. The human operator is conceptualized as an *admittance*—a source of *flows* (*i.e.*, movement), and sink of *efforts* (*i.e.*, forces)—and the VE as an *impedance*—a source of efforts and sink of flows.

At the heart of the passivity controller is α, which is in turn based on the *Passivity Observer* (*PO*). The PO, also known as the parameter *E*_*obsv*_, computes the total energy observed in the system at a given moment as:

$$E_{obsv}\left(n\right)=\Delta T\overset{n}{\underset{k=0}{\sum }}f\left(k\right)v\left(k\right)$$ (1)

where Δ*T* is the sampling time, (*f*) and (*v*) are effort and flow (force and velocity) of the 1 port network at time step *n*. Specifically, *f*_1_ and *v*_1_ are effort and flow for the haptic display, while *f*_2_ and *v*_2_ are for the force computed from the VE computation.

When *E*_*obsv*_(*n*) is negative, the system in losing energy; for positive values it is generating energy. We compute α as:

$$\alpha \left(n\right)=\left\{\begin{array}{cc} -E_{obsv}\left(n\right)/\Delta Tv_{2}{\left(n\right)}^{2},\; & \text{if }E_{obsv}<0.\\ 0,\; & \text{otherwise}. \end{array}\right.$$ (2)

After the VE model is updated, its subsystem forces are recalculated, then passed through α before being passed as commands to the haptic display’s actuators. *f*_1_, the haptic display command force, is computed as the VE force plus the passivity control component (which is acting to siphon excess energy out of the system).

$$f_{1}\left(n\right)=f_{2}\left(n\right)+\alpha \left(n\right)v_{2}\left(n\right)$$ (3)

In this implementation

• If the amount of force exceeds the motor force saturation, we subtract the excess amount and add it to the next time step,
• If the user spends significant time in a mode where the PC is active (dissipating considerable energy to maintain stability), energy will accumulate and the PC will not transmit actuation forces until the user has backed away from the dissipation-requiring usage, allowing the PC to discharge. In practice, we reset the PO’s energy accumulation to zero every 5 s, scenario-tunable scenario or adapted automatically.

#### 4.4.2 Passivity Controller Performance

##### 4.4.2.1 Example 1: Large-Load Coupling

In our first assessment, we examine the performance of our passivity controller for a simple scenario in which the user’s position (*X*_*avatar*_) is “pinned” to a virtual mass as if holding it in their hand. We evaluate performance with two load levels and show how the PCr performs on a large-load coupling.

**FIGURE 8:**
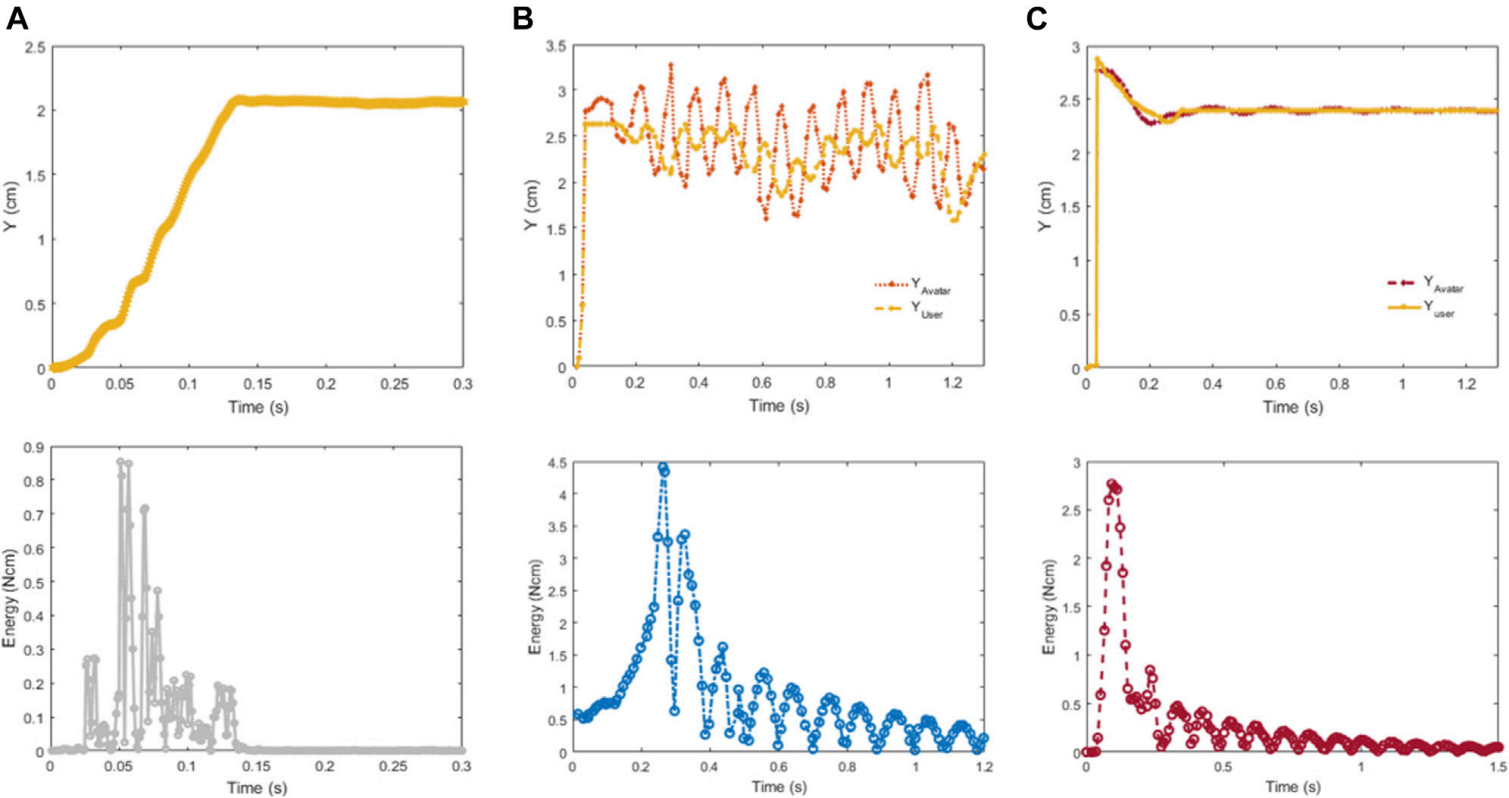
Abrupt movements of varying loads. The position, *i.e.*, *X*_*avatar*_ as it tracks *X*_*user*_ **(upper)** and kinetic energy **(lower)** of the load for **(A)** the original avatar 25 Gram; **(B)** for the avatar with 20 times more mass than the original avatar without the passivity controller, and **(C)** with the passivity controller.

*Virtual Coupling, M = 1X* Figure 8A shows the displacement (upper) and energy output (lower) of the virtual coupling system of Section 4.2.3, *i.e.*, without the PCr. The VC parameters are optimized for this system. Thus, when the user (*X*_*user*_) moves 2 *cm*, *X*_*avatar*_ follows smoothly with no overshoot, achieving steady-state by 150 *ms*. The maximum kinetic energy of PC can potentially reduce performance in a normal case where it is not needed, as it may siphon off system energy even when not necessary, being a conservative approach. Therefore for cases close to the system parameters for which the VC was originally tuned, we switch it off.

*Virtual Coupling, M = 20X* To understand the effect of changing the virtual avatar properties, we investigate a scenario of increasing the mass of the virtual free body being interacted with by 20. Figure 8B shows how the system oscillates following the same user movement. Although the oscillation is bounded by physical damping from the user’s hand, it can become unstable if the user releases the handle. The system kinetic energy peaks at 4.5 *Ncm* then gradually decreases.

*Passivity Control, M = 20X* In Figure 8C, with the PC active with a large mass, the system overshoots by *44%* but converges within 200 *ms* to the desired displacement. System energy peaks at 2.7 *Ncm* and decreases more quickly than in the VC case for the same mass (B).

##### 4.4.2.2 Example 2: User Interacts With a Virtual Mass-Spring System

The previous example showed how PC can handle a large change in the system’s virtual *mass*; how does it do with comparable changes in rendered stiffness as well as the same 20X mass range?

We implement the system as illustrated in Figure 6, where a user draws a mass attached to a spring.

Here, Figure 6B shows a graphical representation of the recognized mode. Our system recognize a zigzag stroke as a spring and rectangle as a mass, and their connection on the sketch as a kinematic connection between them. The experience is similar to pulling on a real spring: force increases as one pulls further. Figure 6B shows the interaction result: as the user pulls down on the spring (change in *X*_*user*_) by around 3 *cm* and then “drops” the force—i.e., stops resisting the haptic display’s applied force—the system applies up to 1.27 *N* of force to restore *X*_*user*_ to its starting position. The system exhibits a damped oscillation, with two sources: 1) the user’s hand and 2) frictions in the haptic display. Here, this is desired behavior faithful to the virtual system dynamics in interaction with the user’s hand damping, not a controller instability.

The graphical representation could optionally be displayed to the user to confirm recognition, and animated as they interact. Drawing and animating could implemented on a co-located tablet screen under the haptic display. In future we plan to investigate impacts of employing the user’s original strokes versus changing them with a cleaner graphical representation, and of animating the diagrams.

The second row in Figure 6 shows the user placing two springs in parallel. The learning concept is that springs in parallel sum to a greater combined stiffness than in series, and the operator should feel a tighter (stiffer) connection. In comparision the previous example, the user should perceive a difference in force for the same displacement: the system supplies up to 2.3 *N* force to the user’s hand for a similar displacement to the single-spring case. As these results show, this system remains stable under passivity control for a doubling of total stiffness in combination with an already-large virtual mass. This mass-spring example can trivially be extended to include a damper (dissipative element). This is energetically less demanding—a virtual damper does not store energy. In general increasing virtual damping (assuming adequate sampling) reduces susceptibility to large impedance variation (Haddadi et al., 2015).

## 5 Discussion

We examine this work’s contributions, and discuss how the PAL approach can be validated and extended.

### 5.1 The Physically Assisted Learning Framework, Guidance and Exposed Needs

We drew on general theories of experiential learning to propose a framework that to help haptic and educational experts work together to leverage physical intuition in an effective learning process. This endeavor needs support: learning technology is notoriously hard to evaluate for efficacy, and get feedback on what is helpful. Despite evidence for the role of physical intuition and embodiment in effective learning, we know far less about how to saliently recreate it in digital contexts. Thus, rather than trying to show directly that haptic feedback helps learning, we built on a proven approach in first 1) accepting that *designing* and *exploring* are powerful supports to learning, then 2) seeing how haptic environments can make these activities more powerful than without them.

#### 5.1.1 Metrics

While we have not yet evaluated our technical demonstrations with students, we will in future choose metrics (as per PAL-inspired goals) to highlight how the activities can be more fluid, engaging, focused, intuitive and insightful than without haptics.

#### 5.1.2 Guidelines for Physically Assisted Learning-Inspired Systems

In applying PAL principles we exposed some key requirements. We made progress in translating these to technical challenges, some of which can be addressed with current state-of-art techniques, and others where we need to further innovate. Here we summarize these, noting that while we have identified one pathway to implement them here (Section 5.2), we hope that others will find more.

1) *Let learners design their own worlds:* PAL (and experiential learning theory generally) indicates that we should lower friction in letting learners (or in some case their teachers) build their environments. This is an old idea—Scratch and its ilk have born rich fruit—but we need this for environments amenable to haptic display for the purpose of accessing physical tuition.
2) *Let learners explore, iterate and compare those worlds with physical feedback:* Exploration should be informative, flexible and fun. Haptic feedback needs to be clear enough to support insights; it must be possible to jump around easily within an environment and try different things; and the whole process should flow, show insights that might not be otherwise available, surprise and delight. This entails a certain quality of haptic display, and curation of environments (*e.g.*, mechanical systems, electrical, hydraulic, chemistry) that while offering broad scope, also guide the learner on a rewarding path.
3) *Moving between designing and exploring and back should be fluid:* When experiential learning is working as it should, learners will generate more questions as they explore, and want to go back, re-design, compare and ask again. If they have to change modalities or undergo a laborious process to alter the environment or compare different examples, this cycle will be inhibited. We wonder if it is worth trying to stay (graphically) on paper while the digital world plays out through the haptic device, for immersion, focus and the intuitiveness of physical drawing; instead of fussing with a GUI.

#### 5.1.3 Support a Broad Space for Experimentation

Instability is a continual risk for haptic force feedback systems, and could quickly turn anyone off as well as obscuring recognizable physical insights. Tightly restricting the explorable parameter space is an unacceptable solution, since it likewise limits the kinds of experiments to be conducted. Passivity control is one approach to a broader range than the methods currently available to novice hapticians *via* libraries.

### 5.2 Technical Proof-of-Concept

In the scope of this paper, we have demonstrated at least one full technical pathway for a system that allows a user to design a haptically enabled system by sketching it on paper while adhering to some basic conventions, then interact with that system haptically—and stably—without changing mode or context across a parameter range of which is larger than typically supported in haptic environments. Its and-stroke recognition supports low-friction *designing*, so users can informally sketch ideas, even alter them. For *exploring,* we identified the inadequacy of the conventional rendering method of virtual coupling given the range of system parameters we need to support, and showed how a more specialized controller (based on passivity theory) could take it to this needed level. We encourage curators of haptic libraries to include passivity control support.

### 5.3 Generalizing to Other Physics Environments: A Bond Graph-Inspired Approach

Our examples demonstrate the ability of a passivity controller to bound a system’s energy and prevent instability across a broad range of simulated system parameters. We did this based on a basic mechanical dynamic system, a mass oscillating with different spring combinations. This step can be translated with relative ease to other systems of interest in science learning.

Bond Graph theory (Paynter, 1960; Karnopp et al., 1990) relates physical domains (*e.g.*, mechanics, electronics, hydraulics) based on energetic concepts of *efforts* and *flows*. This commonality is a means to connect domains, but also translate ideas between them. For our purposes, a physical model developed to represent a mechanical system can be translated with relative ease to an electrical domain.

Bond graphs hold threefold value here. First, technically we can exploit its analogies and representation to translate models and their support to other physical domains. Comparable properties will be relevant. In Bond graphs, springs (mechanical) and capacitors (electrical) are analogs, both idealized to store energy in the same way, as are mass and inductance, dampers and resistors. Table 2, drawn from Borutzky (2011), includes a full list of Bond domain analogies.

**TABLE 2:** Analogy between some conventional physical domains, reproduced from Borutzky (2011).

Domain	Flow	Effort	Compliance	Resistance	Inertance
Electric	Current	Voltage	Capacitor	Resistor	Inductor
Kinetic translation	Velocity	Force	Spring	Damper	Mass
Kinetic rotational	Angular Velocity	Torque	Torsional Spring	Damper	Inertia
Hydraulic	Flow rate	Pressure	Chamber	Valve	Fluid inertia

Second, these analogs provide a language and convention by which to render physical properties haptically: *e.g.*, effort, flow, resistance and inertance can be developed once and re-used in their relation to one another. It simplifies implementation in new domains.

Thirdly and most interesting pedagogically, these analogs are a powerful way to grasp and generalize fundamental relationships in physical systems. The haptic representation will reinforce this Bond-centered generalization, helping learners to transfer their growing knowledge across domains: once they have mastered how the relations between current, voltage, compliance and resistance work in the electrical domain, they should be able to quickly apply them to kinetic or hydraulic systems. It is often the case that a learner feels more comfortable in one domain; they can use this “home” grounding to support their understanding elsewhere.

### 5.4 Future Work: The Path Forward

The progress in this paper documents technical feasibility for a basic implementation of a pen-and-paper interaction approach to interactive, self-driven, exploration-centered physical simulation for the sake of learning and gaining physical insight about ideas. Much work remains before we can claim that the concept is ready for roll-out to students and teachers, far less a typical public high school classroom. We lay out some foreseeable next steps.

#### 5.4.1 Validating the PAL Framework: Establishing Impact on Learning

With a theoretically-grounded framework articulated and technical feasibility demonstrated, the next step is to begin confirming the manner and degree to which it actually supports learning. Validating this framework will require a series of focused studies that empirically evaluate the added value of physicality in *design* and *explore* learning phases as well as fluidity in the transition between them. These studies also need to consider factors such as engagement, ownership of knowledge, self-efficacy, self-confidence, and self-paced learning.

One important investigation is the impact of the specific haptic platform: different characteristics (*e.g.*, workspace size, type of grasp, whether they can write as well as feel, ability to propel themselves autonomously, the nature and magnitude of force feedback they can provide) suit them to different usages and learning-environment implementations. Understanding this is the path to generalizing the PAL frameworkt. Meanwhile, PAL itself provides a structure within which we can identify and respond to the advantages and shortcomings of each device as we seek to support specific learning activities.

#### 5.4.2 Basic Access

First and foremost, building haptic worlds and even accessing them interactively requires considerable expertise and infrastructure. Haptic technology is anything but accessible, and this barrier will need to be breached. As for any educational technology, the principle barriers will be in cost, robustness, versatility and usability or expertise.

*Low-cost Robust and Highly Portable Technology* Lower-cost grounded force feedback devices are becoming more common, but the Haply still costs $300 USD, has a small workspace and is not quite tough enough for a school environment. However, Maker culture has starkly lowered barriers for innovation in this space. With use cases established, we expect to see new GFF device formats be commercialized and toughened. For the ideas described here to succeed, the technology will need to become a commonplace tool. It needs to become a highly portable form factor like a pen or stylus type—such as envisioned in Kianzad et al., 2020.

*Versatility* There will be myriad ways to use physical interaction in a form factor that one can carry around, perhaps first like the nerdly calculator of the 80 s then becoming more ubiquitously useful as a haptically augmented smartphone stylus.

*Usability and Expertise* We have called here for lowering friction and barriers to entry for end-users. This also needs to become more true for system designers, allowing them to participate in development from their home discipline and without engineering expertise—*e.g.*, education experts. Input methods, library construction, support groups and other aspects of development ecosystems will move us in this direction.

*Eventually, Logistical Deployment with Kids* Classrooms are challenging environments. The first point of contact may be science centers and tutoring centers, and potentially on to personal devices (like student calculators) rather than school-supplied technology.

#### 5.4.3 Enhanced Usability, Fluidity and Function

We have described many possible variations and augmentations to a basic implementation, all of which can be explored to discover optimality from logistic and pedagogical standpoints, and inform the direction of further technology development. To name a few (and going beyond innovation in the haptic technology itself):

• CAD-type sketching support at the *design* stage
• More advanced sketch-recognition functions, *e.g.*, setting and modifying simulation parameter values by [re]writing them on paper
• Generating more extensive simulation environments, in multiple domains (*e.g.*, Bond graph extensions)
• Utilizing more sophisticated haptic rendering algorithms as we encounter limits
• Finding good haptic representations for abstract fields such as maths
• Libraries to support educators setting up “design sandboxes”

#### 5.4.4 More Deeply Understanding how Variations Can Maximize Pedagogic Value

Several topics will merit especially deep dives, including longitudinal evaluation; we mention two here.

*Paper vs. Digital Boundaries* When should the system stay entirely paper-based, and when move between graphical and paper—what are the value and limitations of each modality in the situation where for the first time we have a choice about it?

*Supporting Collaboration* How do we best exploit the power of collaborative learning, allowing students to share ideas with peers and jointly work out problems? This sketch-based approach paired with personal or shared haptic devices could be extended to remote learning scenarios with the use of touchscreen tablets. We anticipate that haptic feedback can provide a new vocabulary through which students can communicate.

## 6 Conclusion

A long-awaited promise of ubiquitous computing (Weiser, 1991) is natural access to computational power where and when we need it. Yet, for the most part we remain tied to a small screen and a keyboard or tablet, with constrained space to work, keystroke input, a single viewport with many distractions, and interaction generally on the terms of the device.

In this paper we proposed an approach to support multimodal learning with potential benefits to embodied learning and thinking. It includes a framework drawn from validated theories of experiential learning translated to the physical domain to guide system designers in creating educational systems focused on *designing* and *exploring*; underscoring of the importance of fluid, same-modality movement between these learning phases; demonstrations of the technical feasibility of implementing both idea capture and physical rendering in a pen-and-paper environment; and guidelines and assessment of how to move such a vision forward. We demonstrated these ideas on a fixed small-workspace device, but untethered, infinite workspace grounded force feedback has been prototyped and could be commercially viable given demand.

The present work points to a path away from tethered, disembodied interaction, examining ways to harness the natural fluidity and ease of pen-and-paper interactions and connect them to powerful digital simulation for the purpose of simulation, gaining physical, embodied insight, problem solving and thinking with our sense of touch as well as our heads and eyes. A graphical viewport is not always needed when we have our imagination, a sketchpad and hands to feel.

## Data Availability

The raw data supporting the conclusion of this article will be made available by the authors, without undue reservation.
